# Protocol for establishing a mouse model of bilateral maxillary first molar extraction to study alveolar bone healing

**DOI:** 10.1016/j.xpro.2026.104366

**Published:** 2026-02-16

**Authors:** Jiarui Jiang, Jiayu Mou, Siwei Wang, Jianguo Liu

**Affiliations:** 1 Key Laboratory of Oral Diseases Research, School of Stomatology, Zunyi Medical University, Zunyi 563000, China; 2 Department of Dental Implantology, The Affiliated Stomatological Hospital of Zunyi Medical University, Zunyi, China

**Keywords:** Health Sciences, Model Organisms, Stem Cells, Tissue Engineering, Material sciences

## Abstract

Alveolar bone healing following tooth extraction is a complex biological process. Here, we present a protocol for establishing a reproducible mouse model of bilateral maxillary first molar extraction to study alveolar bone healing. We describe steps for surgery, postoperative care, and tissue collection for downstream genomic, histological, and micro-computed tomography (CT) analyses. We provide practical strategies to minimize root fracture and soft tissue injury, ensuring procedural consistency and high-quality outcomes.

## Highlights

• Steps for minimally invasive extraction of bilateral maxillary first molars in mice
• Instructions for optimizing oral access using rubber bands and cotton balls
• Guidance for intact tooth removal using syringe needles and micro-toothed forceps
• Procedures for collecting alveolar bone tissue for histological and micro-CT analyses

## Abstract (editor highlights)

Publisher’s note: Undertaking any experimental protocol requires adherence to local institutional guidelines for laboratory safety and ethics.

## Graphical abstract

**Figure:**
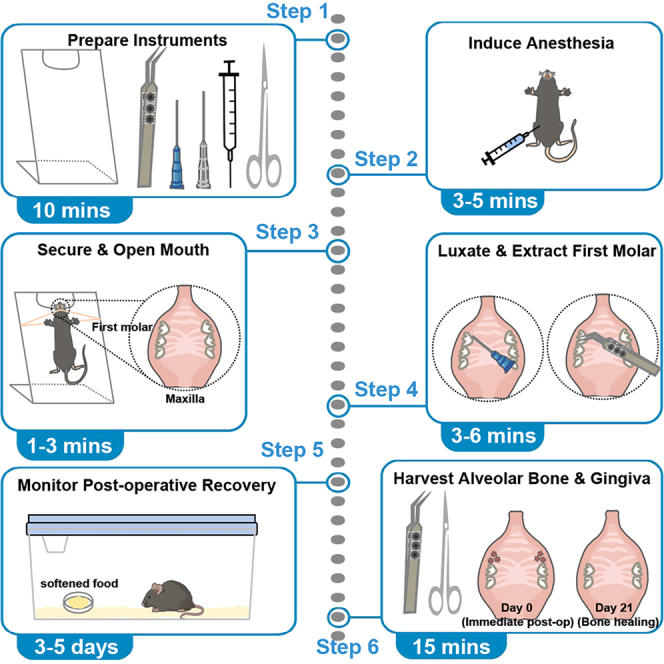

## Before you begin

This protocol establishes a reproducible mouse model of extraction socket healing, suitable for downstream genomic, histological, and micro-CT analyses at defined time points (e.g., immediately after extraction, or at 7, 14, and 21 days post-surgery), as well as for investigating the use of regenerative biomaterials in models of alveolar ridge preservation.

### Innovation

While existing murine tooth extraction models primarily focus on the mandible,^1^ we present a reproducible and minimally invasive protocol for maxillary first molar extraction. Anatomically, maxillary molars are distinguished by the presence of three roots (versus two in mandibular molars) and an alveolar process containing a higher proportion of trabecular (cancellous) bone compared to the denser mandible.^1^^,^^2^ Additionally, maxillary extractions avoid the proximity of the inferior alveolar nerve, eliminating the risk of nerve injury.^3^ Compared with mandibular molars, maxillary molars are more exposed and easier to access, significantly decreasing the risk of iatrogenic complications such as mandibular fracture or accidental injury to the buccal mucosa and tongue.^1^^,^^4^ Consequently, these anatomical and surgical distinctions necessitate extraction strategies specific to the maxilla and offer unique clinical relevance for studying alveolar bone healing.

This protocol incorporates methodological improvements that enhance reproducibility and operator safety throughout the workflow. We utilize a mouse tracheal intubation platform to ensure rigid head stabilization, coupled with the strategic use of two rubber bands to achieve adjustable mouth opening, enabling consistent positioning and optimal oral exposure. To standardize the surgical phase, we provide annotated images marking precise sites for tooth mobilization and detailed instructions for atraumatic tooth extraction. Furthermore, we outline optimized procedures for post-operative care and tissue harvesting, alongside a troubleshooting guide for potential complications. These refinements minimize technical variability and optimize surgical exposure, ensuring reliable downstream histological, micro-CT, and molecular analyses for studies of bone repair, regeneration, and biomaterial-assisted healing.

### Institutional permissions

All animal procedures were reviewed and approved by the Ethical Committee of Zunyi Medical University (Approval No. ZMU21-2506-016). Adult male C57BL/6 mice (6 weeks old) were obtained from the Laboratory Animal Center of Zunyi Medical University. Mice were housed under standard laboratory conditions: a controlled temperature of 22 ± 2 °C, relative humidity of 60%–80%, and a 12- h light/dark cycle (lights on at 08:00, off at 20:00), with ad libitum access to food and water. Softened food was provided post-extraction as described in the Diet section (see below).

***Note:*** Conduct all experimental procedures in strict accordance with institutional guidelines on laboratory safety, animal welfare, and ethical research practices.

### Preparation of surgical instruments, materials, and equipment

**Timing: 10 min (not including autoclave time)**

**Figure 1:**
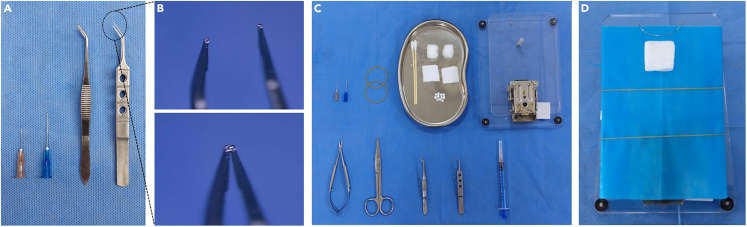
Surgical Preparation (A) Instruments for tooth extraction: 23G and 26G syringe needles (used as dental elevators), blunt-tipped tissue forceps (used as buccal retractors), and toothed ophthalmic micro-forceps(used for tooth grasping and luxation). (B) Magnified view of the toothed tips of the ophthalmic micro-forceps. (C) Complete surgical instrument and supply setup. (D) Preparation of the mouse tracheal intubation platform.
  b. Tissue forceps with blunt, rounded tips to serve as buccal retractors during exposure of the maxillary molar region (Figure 1A).
  c. Ophthalmic micro-forceps with serrated (toothed) tips to grasp and extract the maxillary first molar.^1^ (Figures 1A and 1B).
  d. Sterile surgical drapes to cover surgical table and mouse tracheal intubation platform.
  e. Two rubber bands to depress and stabilize the mandible, serving as an adjustable mouth opener (Figure 1C).
  f. Sterile cotton balls, torn into small pieces (approximately 3–5mm diameter) to be used for hemostasis and cleaning the surgical site (Figure 1C).
  g. Sterile gauze pads to support the neck and protect the cervical spine during surgery (Figure 1C).
  h. Small surgical scissors to incise the oral commissures and dissect soft tissues from the maxilla during sample preparation (Figure 1C).

***Note:*** Sterilize all surgical instruments and materials using a gravity autoclave cycle at 121°C for 15 min,^5^ or according to the sterilization-pouch manufacturer’s instructions. As rubber bands cannot be autoclaved due to heat sensitivity, disinfect them by immersing in 70% ethanol for at least 15 min.^5^ After disinfection, rinse the rubber bands thoroughly with sterile saline or sterile water and allow them to air-dry under aseptic conditions before use.^6^

1. Prepare and sterilize the following instruments and materials to ensure aseptic surgical condition is maintained:
  a. A new set of disposable syringe needles (23G, 25 mm; and 26G, 15 mm) for each mouse to serve as dental elevators (Figure 1A).

***Alternatives:*** Rubber bands can also be prepared by cutting thin elastic strips from a medical-grade latex glove,^1^ which provides an accessible, sterile-compatible substitute when commercial rubber bands are unavailable.

2. Prepare and disinfect the following equipment:
  a. Mouse tracheal intubation platform (20 × 15 × 4.5 cm) with adjustable angulation from 0° to 90° for optimal positioning of the mouse during surgery (Figure 1C).
  b. Feedback-controlled heating pad (18 × 10 cm) to maintain the mouse’s body temperature during surgery.
  c. Connect the stereomicroscope to a power supply and ensure adequate illumination for clear visualization during surgery.

***Note:*** Thoroughly disinfect these equipment with 75% ethanol.

***Alternatives:*** Dental loupes (typically 3.5×–4.5× magnification) serve as an effective alternative to the stereomicroscope and provide sufficient visualization for the extraction procedure.^7^^,^^8^

### Preparation for surgery

**Timing: 10 min**

3. Prepare the surgical area.
  a. Remove any unnecessary equipment from the surgical table.
  b. Disinfect the surgical table with 75% ethanol.
  c. Wear a clean gown, head cover, surgical mask, and sterile gloves before beginning aseptic preparation.
  d. Cover the surgical table with a sterile drape to create a sterile working field.
  e. Open the sterilized surgical tools and arrange them neatly on the sterile drape.
  f. Set up the mouse tracheal intubation platform over the surgical table and adjust it to an angle of approximately 45°–60° to optimize head positioning.
  g. Place the heating pad over the tracheal intubation platform, plug it in, and switch power on.
  h. Place the sterile drape over the heating pad to maintain aseptic conditions.
  i. Place two sterile rubber bands on the draped tracheal intubation platform in advance for later use as mouth retractors during surgery (Figure 1D).
  j. Position a sterile gauze pad directly beneath the stainless-steel wire on the tracheal intubation platform to support the mouse’s neck and protect the cervical spine during surgery (Figure 1D).
4. Anaesthetize the mouse.
  a. Disinfect the abdominal injection site with povidone-iodine before administration.
  b. Administer 1% pentobarbital sodium (50 mg/kg, i.p.) to achieve general anesthesia.^9^^,^^10^
  c. Confirm adequate anaesthesia by performing a toe- or tail-pinch reflex test. If the reflex persists, wait several minutes and retest.

***Alternatives:*** Use any institutionally approved anesthesia protocol appropriate for your facility. A preferred general anesthesia regimen is to induce anesthesia with an intraperitoneal injection of ketamine (100 mg/kg) and xylazine (5–10 mg/kg).^4^^,^^11^ For anesthesia supplementation, administer one-third of the initial ketamine dose only; do not re-administer xylazine.^12^

**CRITICAL:** Continuously monitor the depth of anesthesia throughout the procedure to ensure the absence of reflex responses, with particular attention before performing tooth luxation.

**Figure 2:**
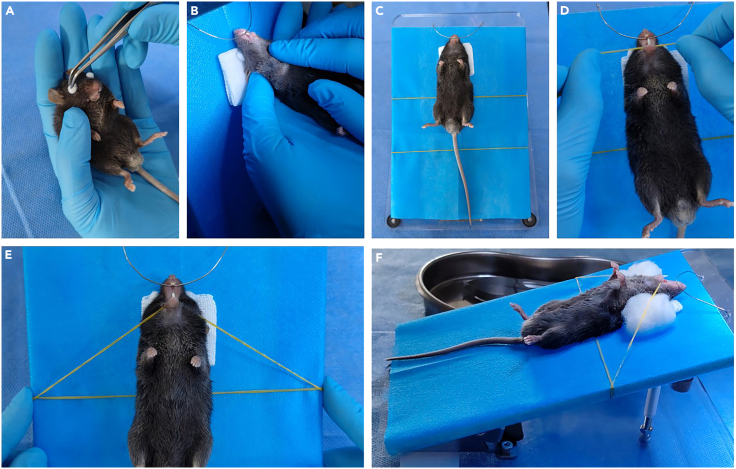
Pre-operative positioning and oral exposure (A) Application of ointment-coated sterile cotton balls to protect the eyes. (B) Positioning of the mouse head to engage the stainless-steel wire. (C) Mouse secured in the supine position with maxillary incisors hooked onto the wire for head stabilization. (D) Placement of the mandibular retractor (rubber band). (E) Adjustment and securing of the retractor to maintain the oral opening. (F) Lateral view of the secured mouse on the tracheal intubation platform, showing cotton balls positioned beneath the rubber bands to cushion mechanical pressure.
6. Administer preoperative analgesia.
  a. Disinfect the dorsal neck injection site with povidone-iodine before administration.
  b. Inject 5.0 mg/kg Carprofen subcutaneously immediately before tooth extraction.

***Note:*** A complete list of all surgical instruments and materials used in this protocol is provided in the **key resources table** (see below).

5. Protect the eyes.
  a. Gently hold the anesthetized mouse in a supine position.
  b. Apply an appropriate amount of ophthalmic ointment onto two sterile cotton balls.
  c. Cover the mouse’s eyes with the cotton balls to prevent dryness and light exposure during surgery (Figure 2A).

## Key resources table

**Table:** 

REAGENT or RESOURCE	SOURCE	IDENTIFIER
**Chemicals, peptides, and recombinant proteins**		

Isoflurane	RWD Life Science Co., Ltd.	Cat# R510-22-10
Pentobarbital sodium	Biohao	Cat# P6031
Lidocaine hydrochloride	Sigma-Aldrich	Cat# 1366013
Carprofen	Tokyo Chemical Industry Co., Ltd. (Shanghai)	Cat# C2701
10% EDTA Decalcifying Solution (pH 7.2)	Leagene Biotechnology	Cat# DD0002
4% Paraformaldehyde Fixative	Biosharp	Cat# BL539A

**Experimental models: organisms/strains**		

C57BL/6J male mice, 6 weeks old	Laboratory Animal Center, Zunyi Medical University	SYXK2021-0004

**Other**		

Stereomicroscope	TM Instrument	TM-77001S
Camera lens	TM Instrument	TM-77001S
HistoCore AUTOCUT	Leica	149AUTO00C1
HistoCore Arcadia C	Leica	14039357263 220V-240V
HistoCore Arcadia H	Leica	14039357259 220V
Tracheal intubation platform for mouse	Tigergene, Nanjing, China	N/A
Autoclave machine	TOMY	SX-300/500/700
Micro-CT scanner	Scanco Medical (Shanghai) Co., Ltd.	VNC-102
Gauze sponges	Yixin Medical Equipment Co., Ltd.	N/A
Sterile cotton balls	Xinxiang Huaxi Medical Supplies Co., Ltd.	N/A
23/26G needle	Sichuan Shuanglu Medical Apparatus & Instruments Co., Ltd.	N/A
Micro-forceps	Suqian Kewo Medical Equipment Co., Ltd.	N/A
Ophthalmic ointment	Baiyunshan Pharmaceutical Co., Ltd.	N/A
75% Ethanol	Guizhou Xinyuan Biotechnology Co., Ltd.	N/A
Povidone-iodine	Shandong Lierkang Medical Technology Co., Ltd.	N/A
Animal warming pad	N/A	N/A

## Step-by-step method details

### Positioning and mouth opening

**Timing: 1–3 min**

This step describes how to position the mouse and open the oral cavity for optimal surgical access.

**Figure 3:**
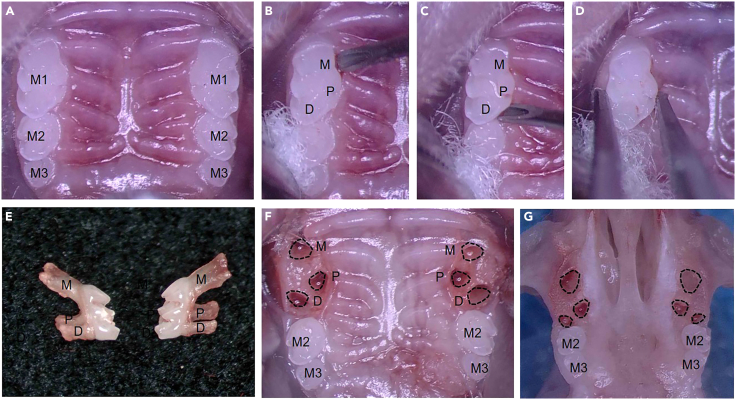
Surgical extraction of maxillary first molars (A) Inspect the position of the maxillary molars under a stereomicroscope. M1, first molar; M2, second molar; M3, third molar. (B and C) Insert the syringe needle tip into the palatal alveolar ridge adjacent to the mesial (M) and distal (D) roots of the maxillary first molar. M, mesial root site; P, palatal root site; D distal root site. (D) Extract the maxillary first molar using a toothed micro-forceps. (E) Examine the bilateral maxillary first molars to confirm intact roots with no signs of root fracture. (F) Inspect the extraction socket. Black dotted lines indicate the positions of the mesial (M), distal (D), and palatal (P) roots. (G) Dissect soft tissues carefully to expose the maxilla and extraction sockets (outlined by dotted lines).

1. Gently hold the anesthetized mouse in a supine position.
2. Slightly tilt the mouse’s head backward to expose the maxillary incisors.
3. Hook the maxillary incisors onto the stainless-steel wire of the tracheal intubation platform to elevate and stabilize the head for the procedure (Figures 2B and 2C).
4. Lubricate both oral commissures with Vaseline using a sterile cotton ball or cotton swab to minimize friction and prevent mucosal injury during rubber band traction.
5. Gently pull up the lower rubber band (positioned closer to the bottom of the platform) and use it to depress the mandible, functioning as the mouth-opening band (Figure 2D).
6. While maintaining tension, hook both ends of the mouth-opening band onto the upper rubber band (positioned beneath the mouse) to anchor it in place (Figure 2E).
7. Adjust the position of the anchored bands to achieve the desired degree of mouth opening (Figure 2E).
8. Place a cotton ball under each rubber band on both sides of the oral commissures to cushion the applied pressure (Figure 2F).
9. Adjust the stereomicroscope view to ensure full visualization of all maxillary molars on the surgical side (Figure 3A).

**CRITICAL:** Ensure the upper rubber band acts as a firm stabilizer for the mandibular retractor. Proper anchoring is essential to prevent loosening or sudden closure of the mouth during the procedure. Maintain a moderate oral opening to ensure sufficient surgical access without overstretching. Avoid excessive force, as overextension of the mandible may dislocate the mandible and result in life-threatening complications.

### Tooth mobilization

**Timing: 1–3 min**

This step demonstrates how a disposable syringe needle is used as a dental elevator to gently loosen the mouse’s maxillary first molar.

10. Moisten a sterile cotton ball with sterile saline and gently wipe the oral cavity to remove any food debris.
11. Soak a small sterile cotton ball in povidone-iodine and gently wipe the right maxillary first molar region for approximately 5-10 s to reduce the local bacterial load.^13^

**CRITICAL:** Ensure cotton balls are damp but not dripping. Before application, gently squeeze the cotton ball or blot it on a dry sterile cotton ball or gauze pad to remove excess fluid. Excess liquid poses a significant risk of flowing into the oropharynx and causing aspiration.

12. Inject 1% lidocaine locally to the mucosa surrounding the maxillary first molar at a dose of 0.4 mL/kg to provide local analgesia.^14^
13. Use tissue forceps with one hand to gently retract the right buccal mucosa.
14. Hold a 26G needle in the other hand and use the tip of the needle to carefully detach the gingiva by severing the supracrestal circumferential periodontal fibers.
15. Slowly insert the needle tip into the palatal alveolar ridge at the mesial (M) root site of the maxillary first molar and gently rotate it (≤45°) to begin loosening the tooth (Figure 3B).
16. Repeat the same maneuver at the distal (D) root site to further loosen the tooth (Figure 3C).
17. Replace with a thicker 23G needle and repeat steps 15 and 16 to further enlarge the space between the first molar and adjacent alveolar bone.
18. Continue gentle rotational movements with the 23G needle until the first molar exhibits evident mobility.
19. Use small sterile cotton balls or cotton swabs throughout the procedure to absorb blood and saliva as needed.

***Optional:*** Place a sterile cotton ball along the buccal vestibule. This acts as a protective barrier during needle manipulation and facilitates immediate hemostasis.

**CRITICAL:** Insert the needle tip at the mesial (M) and distal (D) root sites. Avoid applying force directly at the palatal root site (P), as this risks fracturing the interradicular septum or breaking the palatal root (Figures 3B and 3C).

***Note:*** To master fine instrument manipulation within the limited intraoral space, practice on cadaveric specimens is strongly recommended before performing the live procedure.

### Tooth extraction

**Timing: 1–3 min**

This step describes how to use a toothed micro-forceps to remove the maxillary first molar in mice.

20. Use a toothed micro-forceps to grasp the entire crown of the maxillary first molar, orienting the serrated tips toward the buccal root aspect and extending them beneath the furcation area to securely engage the tooth (Figure 3D).
21. Slightly oscillate the micro-forceps in both buccal and palatal directions while simultaneously applying outward traction to enhance tooth mobility.
22. Slowly extract the first molar by luxating it in the buccal direction with controlled force.
23. Apply sterile dry cotton balls or a small piece of sterile gauze to the extraction socket to achieve hemostasis.
24. Examine the extracted tooth under a stereomicroscope to confirm that all three roots (mesial, distal, and palatal) are intact, that no root fracture has occurred, and that no fragments of alveolar bone remain attached to the extracted tooth (Figure 3E).
25. Inspect the socket for residual root fragments and assess the integrity of the interalveolar septum for signs of fracture (Figure 3F).

**CRITICAL:** Avoid applying rotational force during extraction, as this may cause root fracture.

***Note:*** The extraction of the left maxillary first molar follows the same procedure as the right (Methods video S1).

Methods video S1. Tooth Mobilization and Extraction of Mouse Maxillary First Molar, related to step tooth mobilization and step tooth extraction

### Postoperative care

**Timing: 60 min (surgical recovery) + daily monitoring until stable**

This step facilitates optimal postoperative recovery of the mouse and ensures the consistency of downstream experimental procedures.

26. Recovery.
  a. Place the mouse on a heating pad to maintain body temperature and promote recovery from anaesthesia.
  b. Position the mouse on its side to prevent blood from entering the trachea following tooth removal.
  c. Check the surgical site for any signs of active bleeding.
  d. Continuously monitor the mouse until it fully regains consciousness, confirmed by the return of the righting reflex, then return it to its original cage.^5^
27. Analgesia.

Administer carprofen (5 mg/kg) subcutaneously once every 24 hours for 48 hours post-extraction to alleviate pain.^15^

***Note:*** Perform all injections under strict aseptic conditions.

28. Diet.
  a. Place softened food (e.g., mashed chow) in a sterile Petri dish on the cage floor to facilitate access.
  b. Provide fresh softened food daily for at least 2 days or until the mice resume normal feeding behavior and weight gain.
  c. Supply sterile drinking water and allow ad libitum access.
29. Post-extraction Monitoring.
  a. Record daily food and water consumption for each cage until intake stabilizes.
    i. Calculate the average intake per mouse by dividing total consumption by the number of animals in the cage.
  b. Weigh each mouse once daily at the same time each day (e.g., midpoint of the light cycle) until normal weight-gain patterns resume.
    i. Use the weight recorded on the day of extraction as the baseline for postoperative weight assessment.
      ***Note:*** Mice typically show a reduced rate of weight gain during the first 3–5 days after surgery, after which weight gain usually returns to a stable trajectory.^16^
  c. Monitor mice daily for signs of abnormal behavior or adverse postoperative outcomes (e.g., swelling, bleeding, infection, or signs of pain).
    ***Note:*** Common indicators of pain or distress in rodents include a hunched posture, reluctance to move, decreased food or water intake, vocalization upon handling, aggression, excessive gnawing or consumption of bedding, and poor grooming.^17^
    **CRITICAL:** Immediately and humanely euthanize mice that exhibit persistent signs of pain or continued postoperative weight loss, in accordance with institutional animal welfare guidelines.

### Tissue harvesting

**Timing: 15 min**

This step describes how to harvest alveolar bone tissue at designated time points (e.g., immediately post-extraction, and at days 7, 14, and 21) for downstream analysis. Day 21 is generally considered the time at which the extraction socket is nearly healed in wild-type C57BL/6 mice.^4^^,^^18^

30. Tissue collection.
  a. Euthanize the mouse using carbon dioxide (CO_2_) inhalation followed by cervical dislocation to ensure death.
  b. Place the mouse in a supine position. Use scissors to incise both oral commissures and transect the masticatory muscles attached to the mandible to separate the maxilla from the mandible.
  c. Carefully dissect away soft tissues from the maxilla using curved scissors and forceps, taking care not to damage the extraction socket (Figure 3G).

***Note:*** Alternative euthanasia methods, such as anesthetic overdose with pentobarbital or isoflurane, may be used based on institutional guidelines and experimental requirements.

***Optional:*** For downstream genomic analysis, collect gingival or alveolar bone tissue around the extraction socket. Place tissues in 1.5 mL RNase-free Eppendorf tubes prefilled with RNA stabilization solution. Incubate at 4°C overnight, then transfer to −80°C for long-term storage.

**Figure 4:**
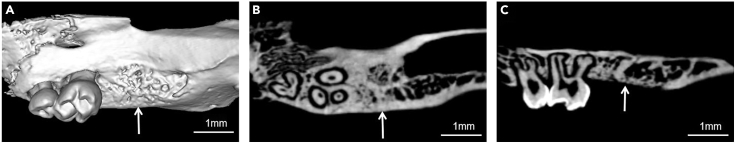
Alveolar Bone Healing at 21 Days Post-Extraction (A) Three-dimensional (3D) micro-CT reconstruction of the alveolar bone. (B) Transverse micro-CT slice image of the alveolar bone. (C) Sagittal micro-CT slice image of the alveolar bone. Arrows indicate the extraction sockets of the maxillary first molars.

***Note:*** Define the region of interest (ROI) as the alveolar socket formerly occupied by the maxillary first molar. Quantify bone surface-to-volume ratio (BS/BV), bone volume fraction (BV/TV), trabecular thickness (Tb.Th), trabecular number (Tb.N), and other relevant indices.

***Note:*** Micro-CT is widely regarded as the gold standard for assessing mineralized tissue microarchitecture in rodent tooth extraction models.^7^^,^^9^^,^^10^^,^^11^^,^^18^^,^^19^ For more detailed guidance on Micro-CT scanning parameters, reconstruction settings, and quantitative analysis workflows, refer to previously published protocols.^2^

31. Micro-CT analysis.
  a. Fix the maxilla in 4% paraformaldehyde for 24 h.
  b. Remove the sample, blot excess fixative with sterile gauze, and place it on the micro-CT scanner stage.
  c. Perform scanning to acquire raw images.
  d. Reconstruct three-dimensional (3D) images using Recon software and perform quantitative analysis using Avatar or other compatible micro-CT software (Figures 4A–4C).

32. Histological analysis^19^^,^^20^^,^^21^
  a. Fix the maxilla in 4% paraformaldehyde for 24 h.
  b. Decalcify the maxilla in 10% EDTA (pH 7.4) at 4°C on a shaker for approximately 1 month.
  c. Replace the decalcifying solution every 2-3 days.
  d. Process the decalcified tissue through graded ethanol (70%, 80%, 95%, and 100%) followed by xylene, using an automated tissue processor.
  e. Embed the maxilla in paraffin with proper sagittal orientation and section at 4-5 μm thickness using a rotary microtome.
  f. Mount sections on adhesive slides for downstream histological and immunohistochemical staining.

***Note:*** Ensure complete decalcification of the alveolar bone before embedding. Gently probe the second or third molar region with a fine needle—easy penetration indicates near-complete decalcification, while persistent resistance suggests that additional decalcification is necessary.

## Expected outcomes

This protocol yields reproducible and minimally invasive extraction socket wounds in mice, enabling downstream genomic, histological, and micro-CT analyses at defined time points of interest.

## Limitations

This protocol provides a practical approach for investigating maxillary bone healing and regeneration in mice. However, several limitations should be considered. First, although a tracheal intubation platform can greatly facilitate stabilization and visualization—particularly for operators with limited experience—it is not strictly required; experienced surgeons may perform maxillary molar extraction on a flat surgical surface with comparable outcomes. Second, operator skill is an important determinant of procedural success, as inadequate experience increases the likelihood of root fracture and soft tissue injury. Finally, while this protocol has been optimized for young adult mice, molar tooth extraction in aged mice (e.g., ≥12 months) presents additional challenges due to age-related increases in cementum deposition.^22^ These age-associated morphological changes elevate the risk of root fracture and increase procedural complexity,^23^^,^^24^ although aged mice can still be used when necessitated by specific experimental designs.

## Troubleshooting

### Problem 1

Inadequate anesthesia (step Anaesthetize the mouse, 4; step-by-step method details, 1-25).

### Potential solutions

Ensure accurate preparation of the anesthetic dose by expelling all air bubbles from the syringe and verifying the injection volume. Inject the anesthetic intraperitoneally at a slow and steady rate. Upon completion, gently rotate the needle and withdraw it slowly to minimize leakage.

Continuously assess reflexes (e.g., pedal withdrawal/toe-pinch). If the mouse retains reflexes or exhibits voluntary movement, administer a supplemental dose according to institutional guidelines. Ensure a surgical plane of anesthesia is re-established before proceeding.

### Problem 2

Physiological distress due to mechanical restraint (step Positioning and Mouth Opening, 5-25).

### Potential solutions

Monitor for physiological stress indicators such as tachycardia, rapid breathing (tachypnea), or gasping, particularly after securing the mandibular retractor. Unlike inadequate anesthesia, these signs may occur even when reflexes (e.g., toe-pinch) are absent.

If these signs occur, immediately release the mandibular retractor (rubber band) to relieve pressure. Pause the procedure and allow the mouse to stabilize and recover a normal respiratory rhythm.

When resuming, re-apply the retractor with less tension, ensuring the airway remains patent.

### Problem 3

**Figure 5:**
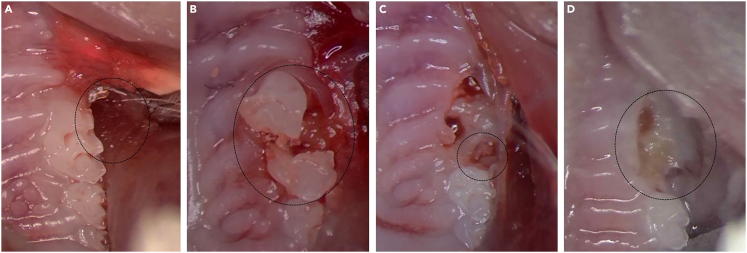
Potential Complications (A) Soft tissue puncture. (B) Crown fracture. (C) Root fracture. (D) Infected extraction socket showing incomplete closure and swelling of the gingival/palatal tissue at 7 days post-extraction.

Soft tissue puncture (step Tooth Mobilization, 13-18) (Figure 5A).

### Potential solutions

Soft tissue injury may occur when sharp-tipped forceps are used to retract the buccal mucosa, particularly by inexperienced operators. To reduce this risk, use forceps with blunt or rounded tips as buccal retractors, or substitute them with a flat, rounded instrument such as a wax-carving tool. Avoid placing the retractor directly against the vestibular groove to minimize mucosal damage.

Additionally, during tooth mobilization, slippage of the syringe needle may lead to buccal or palatal mucosal puncture, causing mild to severe bleeding or potential infection (Figure 5B). Maintain steady and controlled hand pressure on the syringe and avoid applying excessive force when resistance is encountered.

If mucosal puncture results in focal bleeding, apply a sterile, dry cotton ball directly to the puncture site with gentle pressure for several minutes to achieve hemostasis.

### Problem 4

Loosening of the Maxillary Second Molar (step Tooth Mobilization, 16-18).

### Potential solutions

Take special care to avoid inserting the needle between the maxillary first and second molars, as doing so may inadvertently loosen the second molar. If mobility of the second molar is observed, immediately discontinue rotational manipulation and reposition the syringe needle to prevent using the second molar as a fulcrum. Because this loosening does not affect the evaluation of healing at the first molar extraction site, it can typically be considered negligible.

### Problem 5

Crown Fracture (step Tooth Extraction, 20-22) (Figure 5C).

### Potential solutions

Crown fracture of the maxillary first molar may occur during extraction when excessive lateral forces are applied, particularly during buccal-to-palatal rocking with toothed micro-forceps, or when excessive gripping pressure is exerted on the crown. To reduce this risk, grasp the crown as apically as possible to maximize engagement, apply slow and controlled luxation forces, and avoid abrupt lateral movements.

If crown fracture occurs, switch to a 26G needle to further mobilize the remaining crown fragments and attempt extraction of each crown segment along with its corresponding root individually. To minimize the likelihood of this complication, extensive practice on cadaveric mice is strongly recommended prior to performing extractions in live animals.

### Problem 6

Root fracture (step Tooth Extraction, 20-22) (Figure 5D).

### Potential solutions

The mouse maxillary first molar, with its three-root anatomy, is highly susceptible to root fracture—particularly of the distal roots—throughout the extraction procedure. The likelihood of fracture varies with operator skill and animal age; in 6-week-old mice following this protocol, the estimated fracture rate is ∼5%.

Whether to attempt retrieval of the fractured root depends on the location and mobility of the fragment. If the fracture line is flush with the alveolar socket crest and the fragment exhibits slight mobility, carefully enlarge the socket using a 26G needle and remove the fragment with toothed micro-forceps. However, if the fracture surface lies below the socket margin and the fragment is firmly embedded, it is advisable to abandon retrieval to avoid additional trauma.

## Resource availability

### Lead contact

Further information and requests for resources and reagents should be directed to and will be fulfilled by the lead contact, Siwei Wang (wangsiwei22@zmu.edu.cn).

### Technical contact

Technical questions on executing this protocol should be directed to and will be answered by the technical contact, Jianguo Liu (ljg@zmu.edu.cn).

### Materials availability

This study did not generate new unique reagents.

### Data and code availability

This study did not generate datasets or code.

## Acknowledgments

This study was supported by the Science and Technology Fund Project of Guizhou Provincial Health Commission (no. gzwkj2025-442); Guizhou Provincial Department of Science and Technology Support Programme Project: Basic and Clinical Research on Dendrobium officinale Decoction for Anti-Aging Xerostomia Based on a Salivary Gland Organoid Senescence Model (no. Qiankehe Support [2023]); and Rolling Support Program for Scientific Research Platforms and Teams in Provincial Universities, Department of Education of Guizhou Province, China (Key Laboratory of Oral Disease Research of Ordinary Higher Education Institutions in Guizhou Province; no. QianJiaoJi [2022] 025).

## Author contributions

S.W. devised the original protocol. J.J. and J.M. wrote the manuscript and contributed to the technical sections. S.W. reviewed the manuscript, and J.L. edited and finalized the manuscript. All authors read and approved the manuscript.

## Declaration of interests

The authors declare no competing interests.
